# Geostatistical Modeling and Prediction of Rift Valley Fever Seroprevalence among Livestock in Uganda

**DOI:** 10.4269/ajtmh.22-0555

**Published:** 2023-03-06

**Authors:** Carson Telford, Luke Nyakarahuka, Lance Waller, Uriel Kitron, Trevor Shoemaker

**Affiliations:** ^1^ Viral Special Pathogens Branch, Centers for Disease Control and Prevention, Atlanta, Georgia;; ^2^ Gillings School of Global Public Health, University of North Carolina, Chapel Hill, North Carolina;; ^3^ Uganda Virus Research Institute;; ^4^ Department of Biosecurity, Ecosystems and Veterinary Public Health, Makerere University, Kampala, Uganda;; ^5^ Rollins School of Public Health, Emory University, Atlanta, Georgia

## Abstract

Uganda reported cases of Rift Valley fever virus (RVFV) for the first time in almost 50 years in 2016, following an outbreak of Rift Valley fever (RVF) that caused four human infections, two of which resulted in death. Subsequent outbreak investigation serosurveys found high seroprevalence of IgG antibodies without evidence of acute infection or IgM antibodies, suggesting the possibility of undetected RVFV circulation prior to the outbreak. After the 2016 outbreak investigation, a serosurvey was conducted in 2017 among domesticated livestock herds across Uganda. Sampling data were incorporated into a geostatistical model to estimate RVF seroprevalence among cattle, sheep, and goats. Variables resulting in the best fit to RVF seroprevalence sampling data included annual variability in monthly precipitation and enhanced vegetation index, topographic wetness index, log human population density percent increase, and livestock species. Individual species RVF seroprevalence prediction maps were created for cattle, sheep, and goats, and a composite livestock prediction was created based on the estimated density of each species across the country. Seroprevalence was greater in cattle compared with sheep and goats. Predicted seroprevalence was greatest in the central and northwestern quadrant of the country, surrounding Lake Victoria, and along the Southern Cattle Corridor. We identified areas that experienced conditions conducive to potential increased RVFV circulation in 2021 in central Uganda. An improved understanding of the determinants of RVFV circulation and locations with high probability of elevated RVF seroprevalence can guide prioritization of disease surveillance and risk mitigation efforts.

## INTRODUCTION

Rift Valley fever (RVF) is a mosquito-borne zoonotic disease discovered in the 1930s and is caused by the Rift Valley fever virus (RVFV), an RNA virus in the family Bunyaviridae, genus *Phlebovirus*.^1^ Since its discovery, its geographic range has expanded throughout the African continent, surrounding islands, and into the Arabian Peninsula, and has the potential to continue to spread to new continents.^2^

Rift Valley fever has important implications for animal and human health, and can have major economic consequences resulting from the severe disease it can cause among domesticated ungulate livestock.^3^ RVFV infection among livestock occurs via vector-borne transmission from infected mosquitoes, and livestock outbreaks of RVF have resulted in mortality rates of 5% to 20% in adults and 80% to 100% in newborns and developing fetuses.^1^^,^^4^ Human infection occurs from contact with fluids from an infected animal or the bite of an infected mosquito, and is usually less severe than in livestock, such that a significant proportion of asymptomatic or mild infections in humans likely go undetected.^5^^,^^6^ Common symptoms in humans mirror those of influenza or malaria.^7^ Severe symptoms such as hepatitis, retinitis, encephalitis, or bleeding in the stool and nose occur in about 10% of human infections, and approximately 1% of human infections progress to hemorrhagic disease.^1^ The case fatality rate among humans who develop hemorrhagic disease is 50%.^8^

As evident in its wide geographic range, RVFV can be maintained in varying ecological contexts and ecosystems. Large epizootic outbreaks are typically associated with anomalous precipitation and flooding, often correlating with El Niño Southern Oscillation patterns.^9^^,^^10^ Floodwater *Aedes* mosquitoes often drive these outbreaks, as they can transmit the virus vertically to offspring in their eggs, which can survive months or years in dry soil until flooding causes dormant eggs to hatch and transmit the virus by biting susceptible individuals.^11^ Outbreaks are then propagated by numerous mosquito species, such as those from the *Aedes*, *Culex*, *Mansonia*, *Coquillettidia*, and *Eretmapodites* genera.^2^^,^^11^ Past research of environmental predictors of RVF epizootics have found temporal trends in precipitation and vegetation greenness to be highly predictive of outbreaks.^10^^,^^12^ Other important considerations include temperature, elevation, land gradient, soil type, land use, proximity to water sources, and susceptible host density.^13^^–^^15^ Maintenance of RVFV during interepizootic periods can also occur via vertical transmission in *Aedes* mosquitoes under normal amounts of rainfall, causing sporadic spillover into livestock and humans. Some evidence suggests that interepizootic RVFV transmission may also be associated with proximity to or residence in forested areas, proximity to water sources, livestock trade, and nomadic husbandry of ungulate livestock.^16^

**Figure 1.**
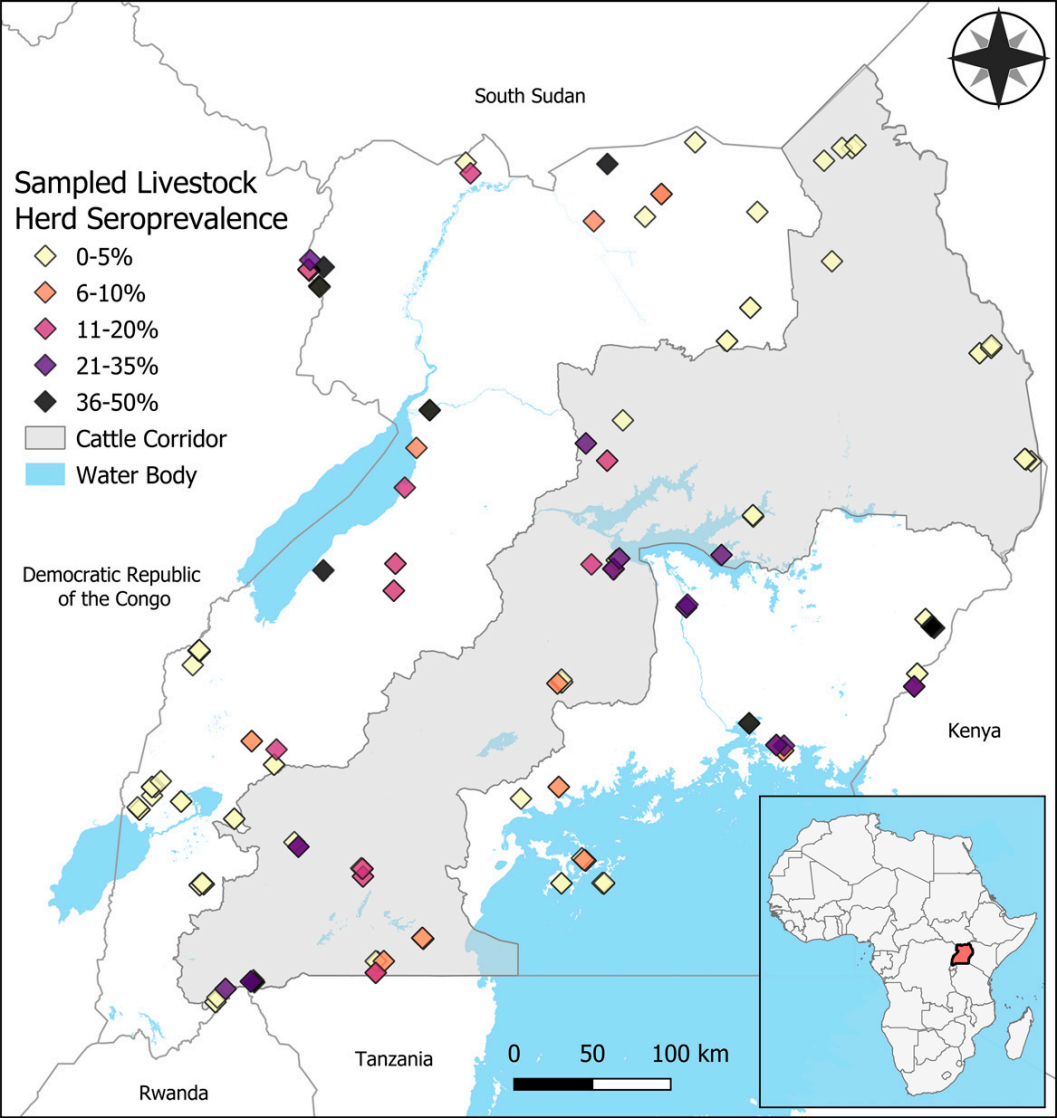
Observed Rift Valley fever seroprevalence among herds of cattle, sheep, and goats in Uganda in 2017.

Until 2016, Uganda had not reported a case of RVF in almost 50 years, when an outbreak resulting in four human infections was identified.^17^ Serosurveys conducted during the outbreak investigation revealed IgG antibodies in 12% of humans and 13% of livestock in the area, whereas only four individuals were found to have active or recent infection via polymerase chain reaction and IgM antibody testing, indicating potentially undetected past exposure to RVFV among RVF-seropositive humans and livestock prior to the onset of the outbreak.^5^ Since 2016, Uganda has continued to identify sporadic transmission that resembles that of interepizootic periods in RVF-epizootic countries. Most outbreaks have occurred in the Cattle Corridor, which stretches from the southwest corner to the northeast corner of the country and is considered the range of most livestock in the country^18^ (Figure 1).

After the detection of RVFV infections in 2016, herds of cattle, sheep, and goats (hereby referred to as livestock) were surveyed and tested for RVFV IgG antibodies in 2017 in 28 districts in Uganda to evaluate the distribution of RVFV in the country. These livestock seroprevalence data were incorporated into a geostatistical model with data on environmental predictors of RVF to generate maps visualizing predicted RVF seroprevalence among livestock in 2017 and the probability that RVF seroprevalence in a given location exceeded 10%; most studies in RVF-endemic countries have shown an average seroprevalence range of 10% to 20%.^19^ Model parameters were then fit to covariate data for the most recent year available to identify locations that recently experienced conditions potentially conducive to RVFV circulation. Regions with high predicted seroprevalence can be targeted for surveillance and increased community education, and prioritized for livestock RVF vaccine distribution.

## MATERIALS AND METHODS

### Livestock sampling data.

Cross-sectional sampling of livestock was conducted between February and August 2017. Blood samples were collected from 3,181 livestock selected from 75 herds of sheep and goats, and 84 cattle herds at 112 sites in 28 of 134 districts in Uganda (Figure 1). Herds selected for sampling were those associated with subsistence farming, and *not* livestock associated with commercial trade networks. The number of animals sampled per species herd ranged from 1 to 60 animals. Sampling targeted the various geographies within the country and border districts where importation of the virus could occur. Samples were collected by a team from the Uganda Virus Research Institute. A comprehensive outline of sampling procedures, testing, and data are reported by Nyakarahuka et al.^20^

### Environmental and population variables.

Suitable environments for RVFV transmission are made up of complex interactions among climatic, geographic, hydrological, and geological variables in combination with anthropogenic considerations such as human and livestock population density, urbanization, agriculture, pastoralism, and land-use change. Geographic data on these variables were obtained on a raster grid of Uganda, and values were extracted at sampling locations to determine their association with RVF seroprevalence in sampled herds. Covariate data were downloaded and analyzed at a spatial resolution of 1 km^2^, and data unavailable for download in this resolution were resampled to match this resolution. The presence of IgG antibodies against RVFV is not time specific, as IgG antibodies can last decades; therefore, it was not possible in this study to identify the time at which sampled livestock were exposed to RVFV.^21^ Because exposure to RVFV can occur at any point in an animal’s life, we developed our model using historical average covariate values that livestock could have experienced during their lifetime. We assumed that the average life span of agricultural livestock in Uganda was approximately 8 years (Food and Agricultural Organization [FAO] Uganda, personal communication); therefore, values of each variable represent annual average values during the 8 years prior to sampling (2009–2016).

Monthly precipitation data were sourced from African Rainfall Climatology, with which we created variables representing annual average of monthly precipitation and the annual coefficient of variation of monthly precipitation (monthly precipitation variability).^22^ Enhanced vegetation index (EVI) data were collected from the National Aeronautics and Space Administration Moderate Resolution Imaging Spectroradiometer (MODIS) terra vegetation indices, with which we created variables representing annual average of monthly EVI, average annual EVI during the rainy seasons, and average annual variability of monthly EVI (calculated as the SD of monthly EVI).^23^ Variability in monthly EVI throughout the year may represent areas that experience greater variation in precipitation or areas with a high density of agriculture, as fluctuations in crop growth and harvest throughout the year cause EVI values ascertained via satellite remote sensing to be less consistent than natural vegetation. The MODIS also provides land cover data with 16 land-type classifications, which were analyzed in seven categories: forest, MODIS 1 to 5; shrubland, MODIS 6 and 7; savannah, MODIS 8 and 9; grassland, MODIS 10; wetland, MODIS 11; anthropogenic, MODIS 12 to 14; and bare, MODIS 15 and 16.^24^ Because transmission may be associated with proximity to a certain land type, we also analyzed variables representing distance in kilometers to each land-type category. Soil data representing percent content of clay, silt, and sand were ascertained from the World Harmonized Soil Database.^25^ Data representing cattle, sheep, and goat density were obtained from the FAO and analyzed for individual species and cumulatively.^26^ Given that most human infections in Uganda since 2016 have occurred within the Cattle Corridor, we also created a binary variable denoting whether a sampled herd was inside or outside the Cattle Corridor. Wild animal density data were not available; therefore, to represent the possibility of RVFV circulation among undomesticated ungulates, we calculated and analyzed a variable representing distance to the nearest wildlife reserve or sanctuary using data from the Open Sustainability Institute.^27^ Hydrological variables were sourced from WorldPop^28^ and the Famine Early Warning Systems Network Land Data Assimilation System,^29^ and included slope, distance to the nearest major waterway, distance to inland water sources, average surface runoff, anomalous surface runoff, and average precipitation anomalies. Annual human population density data were also sourced from WorldPop.^30^ We analyzed a topographic wetness index (TWI) as a measure of susceptibility to natural water runoff that is based on elevation and slope of terrain.^31^ Previous research suggests that environmental changes such as deforestation and land-use change can result in elevated risk for transmission of vector-borne diseases.^32^ To account for these dynamics, we created variables representing the average percent change in annual EVI, EVI seasonality, and log percent increase in human population density per 1 km^2^. Changes in EVI and EVI variability were used to identify potential conversion of forest or natural vegetation to arable cropland, with the assumption that agricultural land has greater variability in EVI values than natural vegetation. Specific data regarding importation of livestock in Uganda or trade of livestock within the country were not available. However, past work has identified high-animal-traffic sites across Uganda, such as livestock markets, illegal border crossings and trade sites, and migration hubs.^33^ To account for proximity to one of these high-animal-traffic sites, we developed a covariate representing distance to the nearest high-animal-traffic site. Data for variables selected for inclusion in the final model were also ascertained for the most recently available complete year, which in our case was 2021.

### Statistical analysis.

Preliminary exploration of covariates associated with RVF seroprevalence was carried out by plotting the relationships between logit seroprevalence of RVF with each variable of interest using piecewise linear splines to capture nonlinear relationships. These relationships were then analyzed using a binomial generalized linear model, and covariates found to be associated with RVF seroprevalence (*α* = 0.05) were then incorporated into a generalized linear geostatistical model (GLGM) using model-based geostatistics.^34^ A cutoff of 0.6 was used when evaluating the correlation between variables to retain in the model. To identify the presence of model overfitting, we evaluated the parameter maximum likelihood estimates in a correlation matrix and used a cutoff value of 0.6 to determine whether linear dependence between any covariates was present. The best-fitting model was selected by identifying the one that reduced uncertainty the most in the cumulative output probabilities of exceeding 10% seroprevalence, as described by Giorgi et al.^35^ The GLGM of the probability that a given sampled animal had positive antibodies for RVF was then implemented in the following framework:

$$\log\left\{p\left(x\right)/\left[1-p\left(x\right)\right]\right\}=\beta_{0}+\beta_{1:n}+S\left(x_{i}\right)+U_{i}\text{,}$$

where *p*(*x*) denotes the prevalence of RVF in location *x*, *β *represents the effects of the specified covariates at each prediction location, *S*(*x*) represents a spatial random effect that follows a Gaussian process with mean zero and variance *σ*^2^, and a Matern correlation function. Model covariates were considered statistically significant using *α* = 0.05. The shape parameter of the Matern correlation function was specified as *k *= 1, based on the profile likelihood for the shape parameter in a logit-transformed linear Gaussian model. Covariance parameters were identified by fitting a semi-variogram of model residuals, which represents the decay in correlation between observations as distance between them increases. The “nugget effect,” *U_i_*, represents the unstructured random variation in the outcome and is included to capture the effects of unmeasured explanatory variables that have little to no spatial structure.

We fit a spatial binomial GLGM using Markov chain maximum likelihood using 200,000 simulations within the PrevMap package^36^ in R version 1.3.1075 (R Foundation for Statistical Computing, Vienna, Austria). Prediction of RVF seroprevalence was carried out on a 1-km^2^ resolution grid of Uganda.

Prevalence predictions were based on covariate values in each prediction location and the correlation structure from the fitted model. Model-based geostatistics uses the spatial autocorrelation of sampled locations from the empirical semi-variogram to weight the influence that nearby sampled locations have on the predicted values in given locations.^37^ During implementation, if a sampled location has an observed seroprevalence of RVF that is greater than expected given the model, a spatial smoothing term, *S*(*x*), serves to increase the predicted values in nearby surrounding locations, and vice versa where the observed RVF seroprevalence is less than would be expected given the model and neighboring values. If a location lies beyond the distance at which observations are correlated (per the semi-variogram), then the prevalence is predicted as the expected value based on the covariates without weights according to surrounding sampling points.

### Spatial prediction.

The model was fit to the covariate values in each prediction location and was used to generate maps of the predicted RVF seroprevalence and the probability that RVF seroprevalence in a given location exceeded 10% for the sampling year (2017). Prediction maps were generated for cattle, and separately for sheep and goats, as seroprevalence was greater among cattle compared with sheep and goats. A single composite prediction map of the weighted average of the cattle predictions and the sheep and goat predictions was also created, where weights were based on density of each animal species according to estimates from the FAO. Although the presence of IgG antibodies cannot identify the time at which an infection occurred, the covariate data we used to estimate seroprevalence is available in near real time and could potentially be used to estimate transmission risk, assuming the predictors we use to model seroprevalence are also predictors of RVF incidence. Therefore, we fit our model to the annual average of monthly covariate values for the year 2021. Predictions for all livestock were again combined based on the weighted average of the species density in each given prediction location, and the difference between predictions in 2017 and in 2021 was calculated to identify locations that experienced conditions in 2021 consistent with elevated RVF seroprevalence as a proxy for potential risk of transmission. Maps were generated using QGIS version 3.1.^38^

### Model validation.

We used Monte Carlo iterations to simulate 1,000 semi-variograms given the fitted model to determine the validity and fit of the spatial correlation structure to the data. A 95% interval defining the variability of the model simulations was used to evaluate whether the model fit the data accurately. The null hypothesis was that the specified model was a good fit to the data. After the simulation, the fitted semi-variogram based on the observed data fell within the 95% interval of variograms estimated from the simulated data, and it was determined that the specified correlation structure fit the data adequately to proceed with prediction.^37^

## RESULTS

### Livestock seroprevalence and model selection.

Among livestock sampled, cattle represented 54% (*n* = 1,732), goats represented 34% (*n* = 1,091), and sheep represented 11% (*n* = 358). Overall seroprevalence among cattle, goats, and sheep was 10.7%, 2.6%, and 2%, respectively. Because seroprevalence and management of sheep and goats were similar, sheep and goats were combined for analysis and comparison against cattle. Among cattle herds sampled, RVFV antibodies were present in 56% (47 of 85), and seroprevalence ranged from 0% to 50%. Among sheep and goat herds sampled, RVFV antibodies were only detected in 18% of herds (19 of 106), and seroprevalence ranged from 0% to 33% (Figure 1). Covariates that had the strongest relationship with RVF seroprevalence that were used in the final model included average annual variability of monthly precipitation, average annual variability of monthly EVI, TWI, log percent increase in human population density, and livestock species.

### 2017 Seroprevalence model and prediction maps.

Covariate maps show that the average annual variability of monthly precipitation was strongest in northeastern Uganda and more stable in the western half of the country, especially between Lake Albert and Lake Victoria in central Uganda. Average annual variability of monthly EVI values was generally greater in northern Uganda, indicating more stable vegetation greenness in the southern half. Spatial trends of the TWI occurred in a vein-like structure throughout the country, being highest in locations where water accumulates, such as near rivers and wetlands. Log percent increase in human population density was greatest in northwestern Uganda, but also in scattered regions throughout the country (Supplemental Figure 1). Analysis of individual covariates in a binomial generalized linear model showed positive linear relationships between logit-RVF seroprevalence and EVI variability, TWI, and log percent increase in human population density, whereas there was a negative relationship between logit-RVF seroprevalence and precipitation variability (Supplemental Figure 1).

Incorporating covariates into a multivariate binomial GLGM, a significant positive association between logit-RVF seroprevalence and EVI variability (*P* = 0.03; 95% CI, 1.98–35.55) was seen, suggesting that livestock RVF seroprevalence tended to be greater in areas that had more variability in vegetation greenness. A significant association was also found with TWI (*P* = 0.02; 95% CI, 0.02–0.27), where RVF seroprevalence was greater in areas susceptible to water runoff and pooling. Livestock RVF seroprevalence tended to decrease in areas with greater precipitation variability (*P* = 0.27; 95% CI, –0.07 to 0.02) and increase in areas with log percent change in human population density (*P* = 0.14; 95% CI, –0.12 to 0.87). Adjusting for environmental variables, seroprevalence among cattle remained significantly greater compared with sheep and goats (*P* < 0.001; 95% CI, 1.31–2.14).

**Figure 2.**
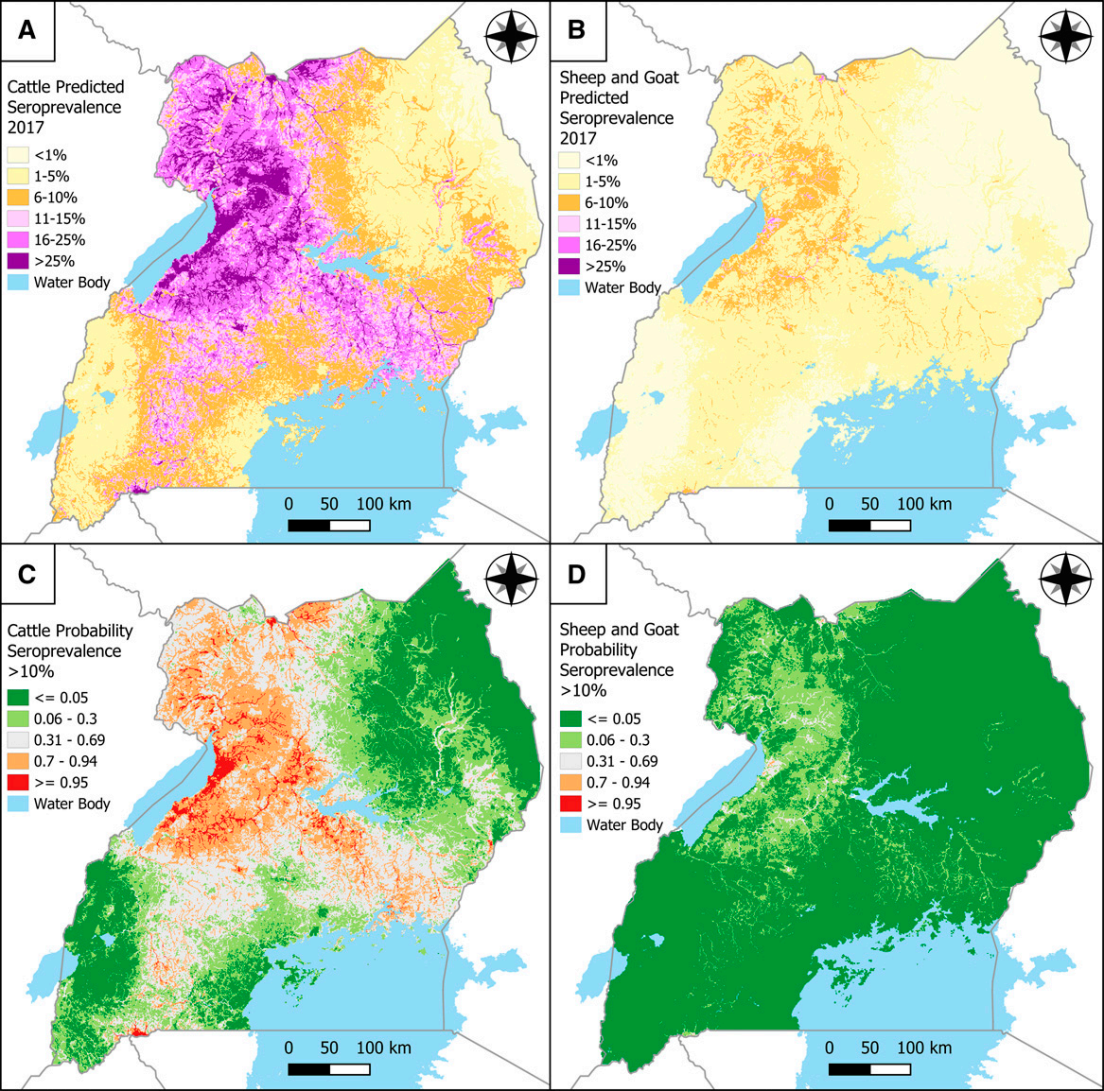
Predicted Rift Valley fever virus (RVFV) seroprevalence across species. Predictions of RVFV among cattle (**A**) and sheep and goats (**B**), and probability that seroprevalence exceeds 10% for cattle (**C**) and sheep and goats (**D**) in the sampling year 2017.

**Figure 3.**
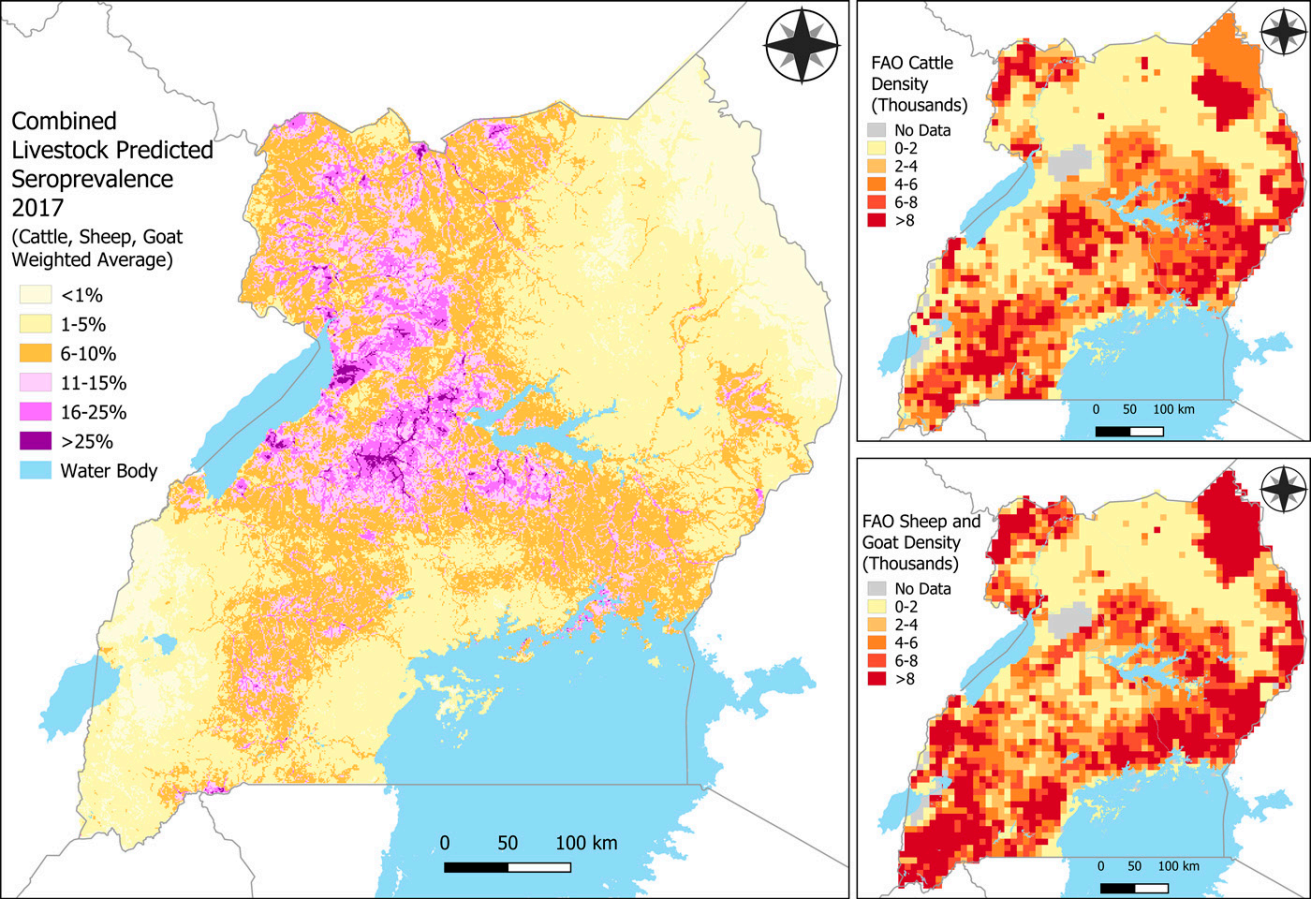
Combined cattle, sheep, and goat density-weighted average prediction map of Rift Valley Fever virus (RVFV) seroprevalence. Weighted average of cattle, sheep, and goat RVFV seroprevalence predictions (**A**), and the estimated density of cattle (**B**) and sheep and goats (**C**) at a spatial resolution of 10 × 10 km from the Food and Agricultural Organization (FAO) that was used for weighting.^26^

Maps of predicted RVF seroprevalence show notably greater predictions of seroprevalence among cattle compared with sheep and goats (Figure 2). Predicted seroprevalence among cattle ranged from 0% to 69%, whereas predicted seroprevalence among sheep and goats ranged from 0% to 31%. The greatest predicted seroprevalence was in northwestern and central Uganda, spreading north from Lake Albert to the border of South Sudan. Seroprevalence was also predicted to be relatively high north of Lake Victoria near the border of Kenya and stretching from the southern border near Rwanda toward Lake Albert along the southern portion of the Cattle Corridor. Elevated predictions of RVF seroprevalence follow a vein-like structure throughout the country consistent with the spatial trend of TWI. The areas with the lowest predicted seroprevalence were in the northeastern quadrant of the country and along the southwestern border of the Democratic Republic of the Congo, where the predicted seroprevalence was generally less than 5%. Data from the FAO on estimated population density of cattle, sheep, and goats show similar spatial distributions across species, although estimated density of sheep and goats was greater than cattle in the southern half of the country (Figure 3). The map of the population weighted average of predictions for cattle, sheep, and goats weighted-average seroprevalence predictions shows the same spatial trend in predictions as seen in the individual species maps, with the focal point of greatest seroprevalence predictions being in central Uganda between Lake Albert and Lake Kyoga (Figure 3).

Maps visualizing the estimated probability that RVF seroprevalence exceeds 10% are presented in Figure 2. Red and orange cells represent areas where there is high probability that RVF seroprevalence exceeds 10%, whereas dark- and light-green cells represent areas where this probability is low. Light-gray cells represent areas of uncertainty, where there is insufficient information to determine with confidence whether the RVF seroprevalence is greater or less than 10%. For cattle, the probability that RVF seroprevalence exceeded 10% was greatest in western and northwestern Uganda along Lake Albert and in central Uganda. Localized locations that also had high exceedance probability were located on the southern border near Rwanda and Tanzania, and along the northern border of South Sudan. For sheep and goats, the probability that RVF seroprevalence exceeded 10% was low across the country, with only small areas of uncertainty that followed a spatial trend consistent with the TWI variable.

Comparing observed herd seroprevalence to the model-predicted seroprevalence in the same locations, we see that predicted seroprevalence was less in many instances, being smoothed by the model after accounting for environmental conditions, the spatial structure of surrounding sampling sites, and the SD associated with fewer observations (Supplemental Figure 2). This is typical behavior for spatial models of disease prevalence. Areas with small sample sizes yield unstable local estimates and the models borrow more information from neighboring sites to stabilize estimates. Most herds with an observed RVF seroprevalence of zero were also those with few animals sampled, and the model-predicted seroprevalence was greater than observed in those locations because the model borrows information from neighboring (and often nonzero) values.

### 2021 Prediction.

Comparing the average monthly covariate values for the year 2021 to average values from 2009 to 2016 (Supplemental Figure 3), monthly precipitation variability in 2021 was greater in central Uganda and west of Lake Victoria. Monthly EVI variability in 2021 was generally less throughout the country, with the greatest values still occurring in northern Uganda. Human population density data were not available for 2021; therefore, we used the most recently available data, which was data for 2020. Human population density in 2020 increased most in northwestern Uganda and near the eastern border with Kenya. The TWI is a constant value derived from elevation and did not change between the two periods.

**Figure 4.**
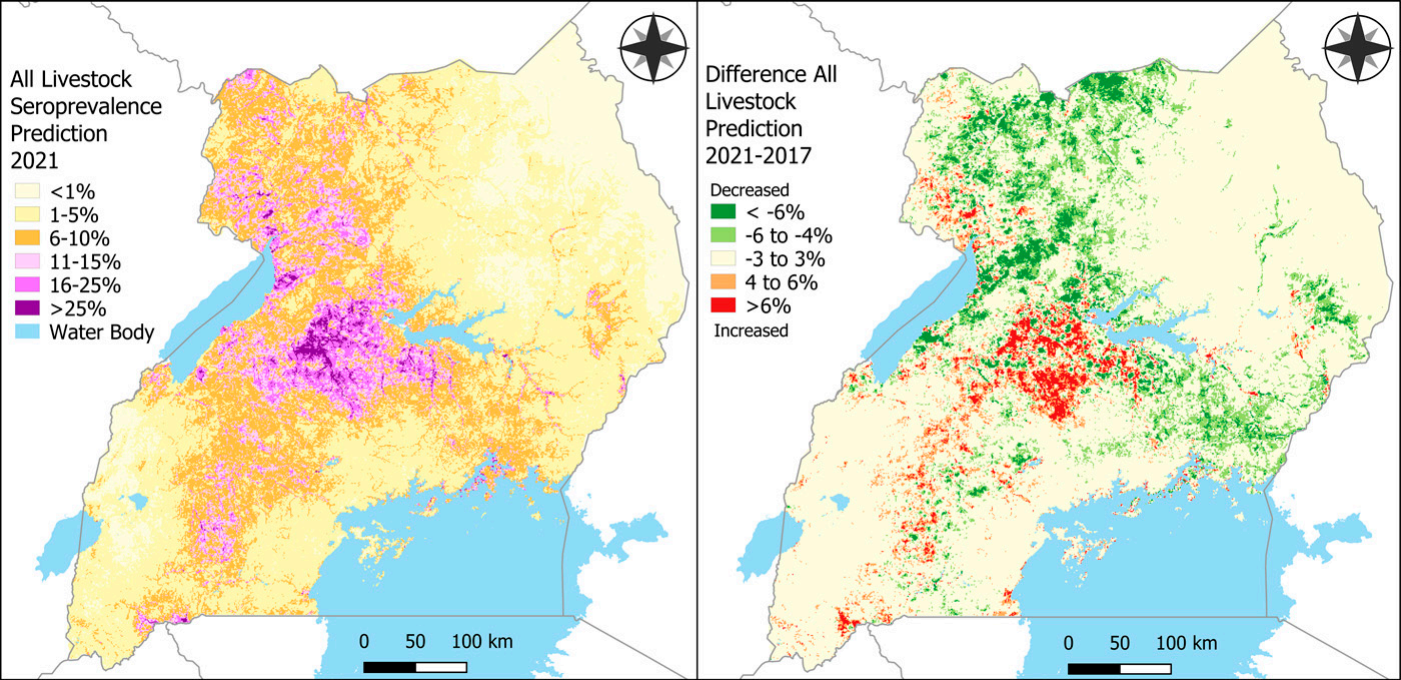
Potential shift in suitable transmission conditions in 2021. The top row represents fitted model parameters from the base model of 2017 seroprevalence to covariate values for the year 2021. These predictions represent potentially suitable conditions for Rift Valley fever (RVF) transmission among cattle (**A**) and sheep and goats (**B**) under the assumption that RVF incidence among livestock is driven by the same factors associated with RVF seroprevalence among livestock, which is what was modeled here. The bottom row represents the difference between the 2017 prediction of RVF seroprevalence (based on annual average of monthly values from 2009–2016) and the 2021 prediction (based on annual average of monthly values for 2021) for cattle (**C**) and sheep and goats (**D**). Red indicates greater predicted values in 2021, suggesting a potentially increased risk of RVF transmission resulting from suitable environmental conditions throughout the year.

The prediction map resulting from fitting the model to the most recently available covariate data shows that the greatest predictions occurred in the central region (Figure 4). The difference in predicted seroprevalence between the 2017 and 2021 predictions show increased predictions in central Uganda and in small patches running through the southern portion of the Cattle Corridor (Figures 1 and 3). Decreases in suitable conditions for transmission may have occurred in northwestern Uganda in 2021.

## DISCUSSION

Our analysis identifies environmental variables associated with livestock RVF seropositivity in Uganda, provides estimates of RVF seroprevalence throughout the country, and identifies areas that recently experienced conditions associated with RVFV circulation. Studies of RVF in endemic countries have found a seroprevalence of 10% to 20%, and our results suggest a high probability of RVFV circulation (≥ 10%) in several regions of Uganda, and lower probability in others.^19^ Overall, predicted RVF seroprevalence was greater among cattle than goats and sheep. The greatest predicted seroprevalence was in the northwestern quadrant of the country, with the focal point being in central Uganda between Lake Albert and Lake Kyoga. Other areas of moderate to high predicted seroprevalence were scattered throughout northeastern Uganda, along the southern Cattle Corridor, and north of Lake Victoria. Several areas where sampling was not done were identified as locations with a high probability of elevated RVF seroprevalence, given the environmental predictors represented in this model and the geographic proximity of sampling locations with elevated seroprevalence. This information can be used to direct future livestock sampling efforts to validate the predictions from this model and, if accurate, can help prioritize surveillance and future mitigation efforts such as livestock vaccine administration campaigns, targeted health-care provider education for earlier identification of cases, and public outreach and education on risk factors for RVF transmission. Directing such efforts to specific geographic locations combined with information ascertained from each covariate included in this model can potentially lead to increased ability to reduce future morbidity and mortality among humans and livestock from RVF.

The region in central and northwestern Uganda, where observed and predicted RVF seroprevalence was greater, is the site of two national parks (Murchison Falls National Park and Paraa National Park) and two wildlife and game reserves (Aswa-Lolim Game Reserve and Ajai Wildlife Reserve). This region does not fall within the Cattle Corridor and was not previously considered a “high-risk” area for RVF outbreaks, and since the reemergence of RVF in Uganda in 2016, acute human or livestock infections have not been identified in this region. Given the relatively lower density of livestock and the lack of identified RVFV infections in this northwestern region, high RVF seroprevalence in these herds may suggest that the maintenance of RVFV in this environment does not depend solely on a transmission cycle between mosquitoes and livestock, but likely also is in some way dependent on the high density of wild game, which typically remain asymptomatic upon infection.^39^ Given that this region also increased in human population density, two possible explanations for elevated RVF seroprevalence in livestock could be the importation of the virus from other RVF-endemic places, or exposure of immunologically naive livestock to RVFV upon introduction into an ecosystem in which RVFV is sustained primarily among mosquitoes and wild game. Further research should evaluate trade networks and seroprevalence of imported livestock in northwestern Uganda to determine where these animals are being exposed to RVFV. Additional analysis should seek to understand the combined role that domesticated livestock and wild game play in RVFV transmission dynamics. Such information could be used to determine the potential effectiveness of interventions such as vector control or vaccination efforts in specific locations.

We found that livestock RVFV seropositivity in Uganda was associated positively with variability in monthly EVI and associated negatively with variability in precipitation (Table 1, Supplemental Figure 1).^10^^,^^12^ One possible explanation for an inverse association with precipitation variability could be that excessive annual variability in monthly precipitation results in either desiccation of mosquito eggs or flushing of larva with too much surface water runoff. The transmission ecology of RVF in Uganda may be different from RVF-endemic countries such as neighboring Tanzania and Kenya, which experience periods of drought followed by precipitation and flooding. Uganda is a country that has a relatively stable climate and receives regular rainfall, and therefore may not be favorable to an RVFV transmission cycle driven by large-scale epizootics. Rather, more consistent monthly precipitation in Uganda may lead to continuous hatching of infected *Aedes* eggs, but rarely on a large scale, and could explain the infrequent, albeit consistent, RVFV transmission. In contrast to precipitation variability, EVI variability was associated positively with RVF seroprevalence, which may result from changes in croplands, which are more variable in “greenness” throughout the year.

**Table 1:** Generalized linear geostatistical model parameter estimates for predictors of RVF seroprevalence

Parameter	Coefficient	Standard error	2.5% CI	98% CI	*P* value
Intercept	−7.89	1.73	−11.28	−4.50	< 0.001
Annual variability of monthly precipitation	−0.03	0.02	−0.07	0.02	0.27
Annual variability of monthly EVI	18.77	8.56	1.98	35.55	0.03
TWI	0.15	0.06	0.02	0.27	0.02
Log human population density % increase	0.37	0.25	−0.12	0.87	0.14
Livestock species (reference: sheep/goats)					
Cattle	1.72	0.21	1.31	2.14	< 0.001
log(*σ*^2^)*	0.14	0.38	−0.60	0.87	–
log(*ϕ*)†	−1.16	0.52	−2.17	−0.15	–
log(*τ*^2^)‡	0.06	0.78	−1.46	1.57	–

EVI = enhanced vegetation index; RVF = Rift Valley fever; TWI = topographic wetness index.

* Variance of the Gaussian process.

† Scale of the spatial correlation.

‡ Variance of the nugget effect.

Increase in human population density was also associated positively with RVF seroprevalence. A national livestock census conducted in Uganda in 2008 reported that 25% of households owned cattle, 40% owned goats, and 9% owned sheep.^40^ Therefore, as human population densities increase, it can be assumed that livestock populations also increase, potentially increasing animal trade and importation of previously infected livestock. However, the owners of only 7% of livestock in our study reported previous herd movement, suggesting that most seropositive livestock were likely exposed in the region in which they were sampled. Aside from increases in susceptible humans and susceptible livestock that humans bring with them, population density change can lead to various environmental changes that lead to enhanced habitat suitability for mosquitoes and elevated risk of RVF outbreaks. Land change resulting from human population growth and the introduction of irrigated agriculture can be beneficial for RVFV circulation because the flooding of fields can cause dormant, infected *Aedes* eggs to hatch, and creates large areas of stagnant water that secondary vectors can use to reproduce.^41^ Land conversion and deforestation resulting from human population growth can also disrupt soil absorption and drainage networks, potentially resulting in flooding.^42^ Although EVI variability and increase in human population density could potentially represent similar anthropogenic changes to the environment that lead to increased RVFV circulation, these variables had a weak correlation, suggesting their encompassment of unique contributions to the dynamics of RVF transmission. Future research should seek to identify the underlying causes of EVI variability in areas of greater RVF seroprevalence in Uganda and its relationship with increases in human population density and mosquito reproduction.

Because our model predicted seroprevalence over space and not specific transmission events, we intend the 2021 prediction map in Figure 4 to be a “risk estimation” that identifies locations that recently experienced optimal environmental conditions for viral circulation, rather than a true prediction of seroprevalence or forecast of disease incidence in 2021. A true prediction of seroprevalence for 2021 would be better suited to incorporate average covariate values from 2014 to 2021, whereas a true forecast of incidence would necessitate a model trained on incidence data. In our case, data on RVF incidence since 2016 in Uganda are still too sparse to train a forecast model accurately for RVF incidence; therefore, this attempt to use annual averages of predictors of livestock seropositivity as a proxy for incidence data should be used with caution. We found that suitable conditions for RVF in 2021 increased in central and southern Uganda, and decreased in northwestern Uganda.

Our analysis is subject to several limitations. First, importation and density of livestock were not accounted for directly because our variables representing livestock density and proximity to high-animal-traffic sites were found to have little to no association with RVF seroprevalence in the model selection process. Likewise, no association was found between RVF seroprevalence and whether a herd was sampled inside or outside the Cattle Corridor. Therefore, although livestock are at greatest risk of RVF, livestock density and importation were not used directly to predict RVF seroprevalence across the country. Livestock density may be accounted for indirectly within the variables representing increases in human population density and EVI variability, as urbanization and agriculture may be associated with livestock presence, pastoralism, and trade. Second, seroprevalence surveys are unable to account for the strong temporal trends associated with RVF outbreaks. We evaluated variables such as average EVI during the rainy seasons, average surface runoff and precipitation anomalies, and variability of precipitation and EVI, and found that variables measuring this variability have the strongest association with our sampling data. To correlate specific weather events with RVF seroprevalence, secondary testing could be used to measure changes in RVF seroprevalence in relation to weather events between sampling periods. All covariates were available through 2021, with the exception of change in human population density, which was available through 2020. Therefore, our predictions of seroprevalence were made using the most recently available human population density data, which may not represent the population dynamics in 2021. In addition to the previously discussed limitations of estimating potential risk of transmission in 2021, we also assume that the covariates explain all of the temporal variation in RVF transmission, and what is unexplained by the covariates represents only spatial residual correlation and not spatiotemporal residual correlation. This assumption cannot be tested, given that longitudinal data are not available at this time, and future research should aim to gather adequate data to develop a model that accounts for space and time.

## CONCLUSION

Our analysis estimated RVF seroprevalence in Uganda in 2017 by fitting a geostatistical model to livestock sampling data and environmental predictors. The greatest predicted seroprevalence was in the central and northwestern quadrant of the country, surrounding Lake Victoria, and along the southern half of the Ugandan Cattle Corridor. The lowest predicted seroprevalence was in the northeastern corner of the country, which encompasses the northern portion of the Cattle Corridor. The variables found to be the best predictors in a model of RVF seroprevalence included annual variability of monthly precipitation and EVI, TWI, log percent increase in human population density, and livestock species. A prediction map for 2021 was generated using the most recently available environmental data to estimate geographic locations that may have recently experienced increased risk for RVF transmission, finding that the predictions increased in parts of central and southwestern Uganda. These results can be used to guide the prioritization of surveillance and risk mitigation efforts such as RVF vaccine distribution, and community and health-care provider education regarding RVFV transmission prevention and case identification.

## Supplemental files


Supplemental materials
