# Supplementary material for: Geostatistical Modeling and Prediction of Rift Valley Fever Seroprevalence among Livestock in Uganda
Source: Am J Trop Med Hyg. 2023 Mar 6;108(4):712–21. doi: 10.4269/ajtmh.22-0555 (PMC10076992; doi:10.4269/ajtmh.22-0555)

Supplemental Figures: **Geostatistical modeling and prediction of Rift Valley fever seroprevalence among livestock in Uganda**

Carson T. Telford, MPH1,2 (ORCID ID: 0000-0002-2778-2597)*; Luke Nyakarahuka, PhD3,4; Lance A. Waller, PhD5; Uriel Kitron, PhD5; Trevor Shoemaker, PhD, MPH1

1. Viral Special Pathogens Branch, Centers for Disease Control and Prevention

2. Gillings School of Global Public Health, University of North Carolina

3. Uganda Virus Research Institute

4. Department of Biosecurity, Ecosystems and Veterinary Public Health, Makerere University, Kampala Uganda.

5. Rollins School of Public Health, Emory University

Supplemental Figure 1. Distribution maps of predictors of RVF livestock seroprevalence and regression lines of their linear associations with logit-seroprevalence of RVF. Geographic distributions of covariates including precipitation seasonality (A), EVI seasonality (B), topographic wetness index (TWI) (C), and percent change in log human population density (D)(left column), and the linear association between each covariate and logit-RVF seroprevalence (right column).

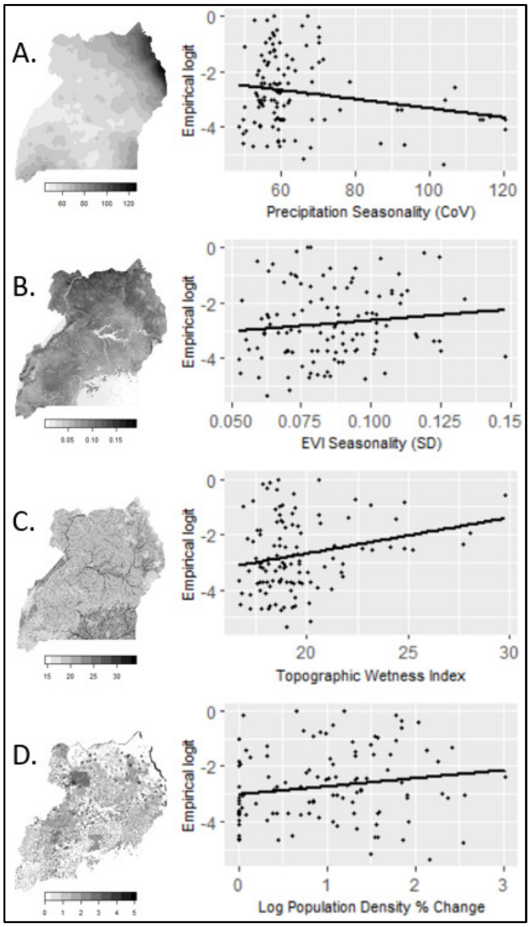

Supplemental Figure 2. Model predictions compared to observed herd seroprevalence. Model predicted Rift Valley Fever seroprevalence among Cattle herds (A) and Sheep and Goat herds (B) compared to what was observed during sampling in 2017.

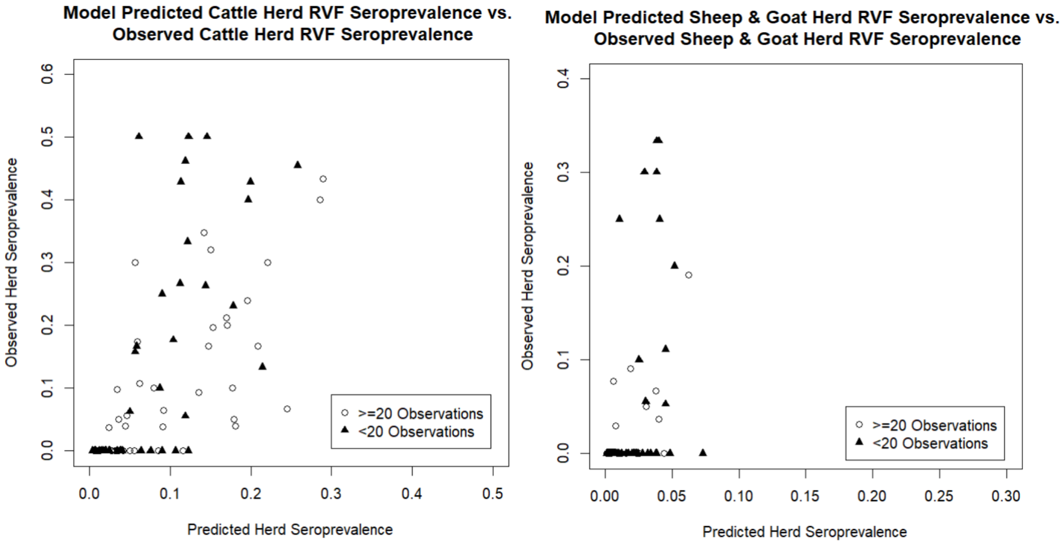

Supplemental Figure 3. Comparison of model covariate values for the 2009-2016 time period compared to 2021. The left column represents data from years 2009-2016 while the right column represents data for the year 2021. Human population density data from 2020 was used to estimated 2021 risk due to unavailable data for year 2021 at time of this analysis.

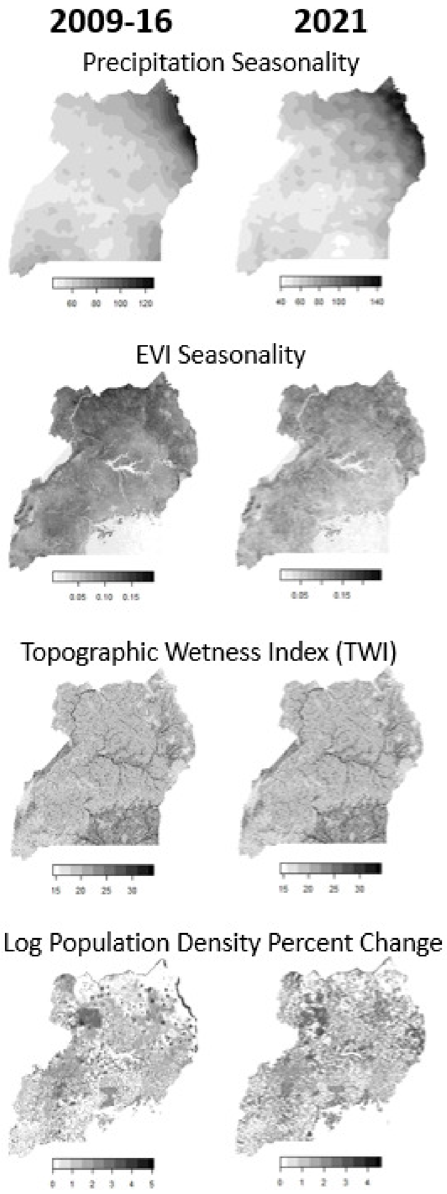

Supplement: Supplementary file 1 [file tpmd220555.SD1.pdf]
